# Disrupted White Matter Functional Connectivity With the Cerebral Cortex in Migraine Patients

**DOI:** 10.3389/fnins.2021.799854

**Published:** 2022-01-13

**Authors:** Zhaoxia Qin, Huai-Bin Liang, Muwei Li, Yue Hu, Jing Wu, Yuan Qiao, Jian-Ren Liu, Xiaoxia Du

**Affiliations:** ^1^School of Psychology, Shanghai University of Sport, Shanghai, China; ^2^Department of Medical Imaging, The Affiliated Hospital of Yangzhou University, Yangzhou, China; ^3^Department of Neurology, Jiuyuan Municipal Stroke Center, Shanghai Ninth People’s Hospital, Shanghai Jiao Tong University School of Medicine, Shanghai, China; ^4^Clinical Research Center, Shanghai Jiao Tong University School of Medicine, Shanghai, China; ^5^Vanderbilt University Institute of Imaging Science, Nashville, TN, United States; ^6^Department of Radiology and Radiological Sciences, Vanderbilt University Medical Center, Nashville, TN, United States

**Keywords:** migraine, BOLD, white matter, functional connectivity, gray matter

## Abstract

**Background:** In attempts to understand the migraine patients’ overall brain functional architecture, blood oxygenation level-dependent (BOLD) signals in the white matter (WM) and gray matter (GM) were considered in the current study. Migraine, a severe and multiphasic brain condition, is characterized by recurrent attacks of headaches. BOLD fluctuations in a resting state exhibit similar temporal and spectral profiles in both WM and GM. It is feasible to explore the functional interactions between WM tracts and GM regions in migraine.

**Methods:** Forty-eight migraineurs without aura (MWoA) and 48 healthy controls underwent resting-state functional magnetic resonance imaging. Pearson’s correlations between the mean time courses of 48 white matter (WM) bundles and 82 gray matter (GM) regions were computed for each subject. Two-sample *t*-tests were performed on the Pearson’s correlation coefficients (CC) to compare the differences between the MWoA and healthy controls in the GM-averaged CC of each bundle and the WM-averaged CC of each GM region.

**Results:** The MWoAs exhibited an overall decreased average temporal CC between BOLD signals in 82 GM regions and 48 WM bundles compared with healthy controls, while little was increased. In particular, WM bundles such as left anterior corona radiata, left external capsule and bilateral superior longitudinal fasciculus had significantly decreased mean CCs with GM in MWoA. On the other hand, 16 GM regions had significantly decreased mean CCs with WM in MWoA, including some areas that are parts of the somatosensory regions, auditory cortex, temporal areas, frontal areas, cingulate cortex, and parietal cortex.

**Conclusion:** Decreased functional connections between WM bundles and GM regions might contribute to disrupted functional connectivity between the parts of the pain processing pathway in MWoAs, which indicated that functional and connectivity abnormalities in cortical regions may not be limited to GM regions but are instead associated with functional abnormalities in WM tracts.

## Introduction

Migraine, a severe and multiphasic brain condition, is characterized by recurrent attacks of moderate-to-severe headache. The condition is usually accompanied by multiple sensory symptoms such as nausea, vomiting, and hypersensitivities to olfactory, visual, auditory, and somatosensory stimuli (Goadsby et al., 2017). Accumulated neuroimaging studies have found that migraine patients exhibit functional and structural abnormalities in a variety of cortical (Zhang et al., 2016, 2017a,b; Skorobogatykh et al., 2019), subcortical pain-processing regions (Qin et al., 2019, 2020a; Skorobogatykh et al., 2019; Younis et al., 2019; Kullmann and Veit, 2021) and white matter fiber tracts (Akatsuka et al., 2008; Ashina et al., 2021), that are often referred to as being part of the pain processing pathway. The pain processing pathway is believed to be involved in integrating all sensory, cognitive, and affective responses to pain, and it becomes active during nociceptive processing (Goadsby et al., 2017). Previous studies found white matter microstructure alterations in migraine patients by diffusion-tensor imaging (DTI) technology (Qin et al., 2019); however, function-related signals within the white matter (WM) tracts and functional interactions between the WM fibers and gray matter (GM) regions remain unclear.

Functional magnetic resonance imaging (fMRI) based on blood oxygenation level-dependent (BOLD) contrast has been widely used to detect changes in neural activity in the GM but can also identify similar meaningful signals in the WM (Gore et al., 2019). Considering the unique characteristics of white matter signals, some studies have observed reliable WM activation by using appropriate techniques (Mazerolle et al., 2013; Gawryluk et al., 2014). Recently, researchers have reported that BOLD signals in the WM convey rich functional information reflecting neural activity and connectivity, at rest or during tasks (Ding et al., 2016; Li et al., 2019). For example, resting-state and task-evoked magnetic resonance imaging (MRI) signals in the WM are modulated by neural activity in the GM tissues to which they connect (Wu et al., 2017). Ding et al. (2018) have recently shown that BOLD signal waveforms in stimulus-related WM pathways are synchronous with the applied stimulus, and the signal in the WM pathway shows apparent task specificity. Furthermore, the correlation of resting fMRI signals can detect WM bundle and specific GM functional areas, and these correlations are regulated by functional loading (Ding et al., 2018).

In attempts to understand the migraine patients’ WM fibers and GM regions functional architecture, a novel method was used to investigate function-related signals within the WM tracts and functional interactions between the WM fibers and GM regions in the current study, and this method has been used in some brain diseases research (Gao et al., 2020; Huang et al., 2020; Lin et al., 2021). BOLD fluctuations in a resting state exhibit similar temporal and spectral profiles in both WM and GM, and their relatively low frequency (0.01–0.1 Hz) signal powers are comparable (Ding et al., 2016). Thus, it is feasible to explore the functional interactions between WM tracts and GM regions in migraine. In the current study, we used resting-state fMRI to explore the functional connectivity between WM fibers and GM regions in patients with migraines without aura (MWoA), which can illustrate the importance of WM brain function. We hypothesized that the resting-state functional connectivity between WM fibers and GM regions in patients with MWoA may change significantly; the altered functional connectivity may be associated with patients’ clinical data such as headache frequency, pain intensity, duration of disease, headache impact test (HIT-6) score, and migraine disability assessment scale (MIDAS).

## Materials and Methods

### Subjects

The Independent Ethics Committee approved this study of Shanghai Ninth People’s Hospital [Project No. (2016) 01] and the Human Research Committee of East China Normal University (Project No. HR2015/03011). All procedures were performed following the Declaration of Helsinki, and all subjects signed written informed consent. Patients were diagnosed with MWoA by neurologists at the outpatient clinic of the Department of Neurology at Shanghai Ninth People’s Hospital, according to the International Classification of Headache Disorders 3rd edition criteria (IHS, 2018). This study recruited 48 patients with MWoA (age: 38.1 ± 10.4 years) and 48 age and gender-matched healthy controls (HCs) (age: 39.0 ± 11.0 years) to perform the MRI experiments.

We also collected the age, gender, attack duration (hours), frequency (times/month), and pain intensity (VAS) of migraine patients. Patients also completed the MIDAS and HIT-6 to assess their headache-related disability accurately. Each patient filled out a questionnaire about the prevalence and traits of the headache, medications, and other diseases. None of the patients reported that they took preventive medicines or suffered from chronic migraines.

All patients with MWoA included in this study underwent an MRI scan during the interictal period. They had no headache 2 days before or 1 day after the MRI scan, and they did not have discomfort or migraine attacks during the MRI scan. The HCs had no headache or chronic pain disorder in the past year, and HCs with migraine previously and a family history of headaches were excluded. Also, immediate relatives of HCs did not have migraines or other headaches. Finally, the following exclusion criteria were followed for both the patient group and the control group: left-handedness, drug abuse, any neurological or psychiatric disease, metabolic disease (e.g., diabetes), or cardiovascular disease based on clinical examination and structured interviews. The demographic and clinical data are shown in Table 1.

**TABLE 1:** Demographic data and clinical scores of the MWoA group and control group.

	Migraine group	Control group	
	(Mean ± SD)	(Mean ± SD)	*P*-value
N	48	48	1
Gender (male)	29.2%	29.2%	1
Age (years)	38.1 ± 10.4	39.0 ± 11.0	0.68
Disease duration (years)	8.5 ± 6.0	−	−
Attack duration (hours)	15.3 ± 18.4	−	−
Attack frequency (times/months)	3.8 ± 3.3	−	−
Pain intensity VAS score	7.2 ± 1.8	−	−
MIDAS score	23.1 ± 28.6	−	−
HIT-6 score	60.4 ± 12.0	−	−

*VAS, visual analog scale; MIDAS, migraine disability assessment scale; HIT-6, headache impact test;−, No data The P-values are based on two-tailed t-tests.*

### Magnetic Resonance Imaging Acquisition

MRI imaging data were collected using a 3.0 Tesla Siemens Trio Tim system equipped with a 12-channel head coil. The subjects were instructed to stay still, close their eyes, and stay awake and relaxed. During the scanning, all subjects wore Siemens earphones to reduce the impact of noise. The whole MRI scan lasted about 21 min, including 2–3 min of adaptation and localization scan. The high-resolution T1-weighted images were acquired by a 3-dimensional magnetization-prepared rapid-acquisition gradient-echo pulse sequence. The main scanning parameters were as follows: echo time = 2.34 ms, repetition time = 2,530 ms, inversion time = 1,100 ms, matrix size = 256 × 256, field of view = 256 mm × 256 mm, flip angle = 7°, number of slices = 192, slice thickness = 1 mm, sagittal orientation, scanning time: 6 min 3 s. The high-resolution T2-weighted images were acquired by SPACE sequence. The main scanning parameters were as follows: echo time = 408 ms, repetition time = 3,200 ms, matrix size = 256 × 256, field of view = 256 mm × 256 mm, averages = 2, number of slices = 192, slice thickness = 1 mm, sagittal orientation, scanning time: 4 min 59 s. The images in the resting-state were generated by a T2*-weighted gradient-echo echo-planar imaging (EPI) pulse sequence. The parameters were as follows: transverse orientation; 210 volumes; flip angle, 90°; 33 slices of 3.5 mm thickness; repetition time, 2 s; echo time, 30 ms; the field of view, 220 mm × 220 mm; matrix size, 64 × 64; scanning time: 7 min 4 s. There is no chemical reagent in the whole process, and the subjects can cooperate well. The noise of the general MRI scanning room is about 77.9 dB. The EPI sequence is the loudest, about 110 dB. The subjects were fitted with Siemens binaural active noise reduction headphones (>10 dB), and the noise was reduced to less than 100 dB.

### Resting-State Data Preprocessing

Data Processing Assistant for Resting-State fMRI (DPARSF)^1^ was used to preprocess resting-state fMRI data, and Statistical Parametric Mapping (SPM12)^2^ was used for functional data analysis. The first 10 volumes were discarded for signal equilibrium, and slice-timing was corrected. The images for each subject were motion-corrected, if the image’s translation was > 2 mm or their rotation was > 2° in any direction have been discarded. We utilized the Friston 24-parameter model to regress out the head motion effects from the realignment. Then individual structural images were coregistered to the mean BOLD image. The transformed T1-weighted images were segmented into GM, WM, and cerebrospinal fluid regions and were normalized to the Montreal Neurological Institute space. Based on the same deformation field, EPI images were then normalized to the Montreal Neurological Institute (MNI) space using the Diffeomorphic Anatomical Registration Through Exponentiated Lie Algebra (DARTEL) tool. We performed temporal bandpass filtering (0.01 < f < 0.1 Hz) to reduce the influence of low-frequency drift and high-frequency respiratory and cardiac effects. Nuisance signals from the cerebrospinal fluid and linear trends were regressed out. The white-matter signals were not regressed out because this could eliminate signals of interest. Spatial smoothing was performed with a 4 × 4 × 4 mm^3^ full-width half-maximum Gaussian kernel. Finally, time series in the preprocessed BOLD images were normalized into unit variance voxelwise.

### Resting-State Correlations Between the White Matter and Gray Matter

Resting-state correlation analyses were restricted to the GM and WM regions. According to Brodmann’s definition, GM region masks were constructed by the PickAtlas tool (Maldjian et al., 2003), including 82 regions (41 cortical brain regions in each hemisphere). At the same time, WM tract masks from the JHU ICBM-DTI-81 WM atlas (Mori et al., 2008) were used to segment each subject into 48 bundle regions (21 bundles in each hemisphere and 6 commissure bundles). All bundle masks were further constrained within each subject’s whole-brain WM mask with a high threshold of 0.8 to avoid signal contamination by nearby GM areas. Pearson’s correlations between the mean time courses of each WM bundle and GM regions were computed for each subject. Fisher’s r to z transformation was performed for further statistical analysis. These correlation coefficients were averaged among subjects in the group to obtain a group-level matrix representing the relationship between white matter and gray matter networks. The pipeline of fMRI data preprocessing and computing functional connections between each of the WM bundles and GM regions are presented in Supplementary Figure 1.

**FIGURE 1:**
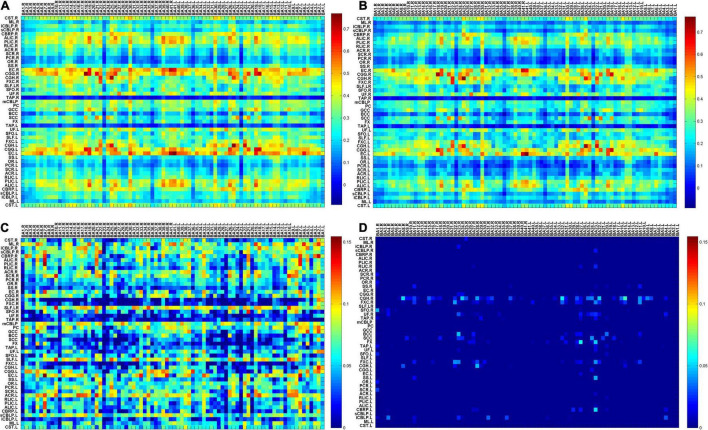
**(A)** The map of average temporal correlations of the HC group between BOLD signals in 48 WM bundles and 82 GM regions; **(B)** the map of average temporal correlations of the MWoA group between BOLD signals in 48 WM bundles and 82 GM regions **(C)** the map of average temporal correlations of the HCs minus those of the patients with MWoA between BOLD signals in 48 WM bundles and 82 GM regions; **(D)** the map of average temporal correlations of the patients with MWoA minus those of the HCs between BOLD signals in 48 WM bundles and 82 GM regions. Each element is the correlation coefficient (CC) between a pair of WM and GM regions averaged across each group. BA denotes the Brodmann area, and abbreviations for the WM bundles are listed in Supplementary Table 1.

To visualize the differences in functional connections between migraine patients and healthy people, the average temporal correlations in a resting state between BOLD signals in 82 GM regions and 48 WM bundles are shown in Figure 1, including the maps of average temporal correlations of the HC group or MWoA between BOLD signals, the maps of average temporal correlations of the HC minus the MWoAs or MWoA minus the HC between BOLD signals. Each element is the correlation coefficient (CC) between a pair of WM and GM regions averaged across each group. The abbreviations for the WM bundles are listed in Supplementary Table 1.

The two-sample *t*-test was conducted on the z-score of the Pearson’s CC to compare the differences between the MWoA and HC groups in GM-averaged CC of each bundle and WM-averaged CC of each GM region. Two-sample *t*-tests were used to test the significance of the group differences with a false-positive correction *p* < (1/N) (regarding the WM-averaged CC of each bundle, *N* = 82; regarding the GM-averaged CC of each WM region, *N* = 48), which is equivalent to the expectation of less than one false-positive regional result per 48 WM bundles or 82 GM regions at this threshold. False discovery rate correction (FDR) was used to correct for multiple comparisons. At the same time, *P* < 1/N for multiple testing corrections (Lynall et al., 2010; Fornito et al., 2011; Cao et al., 2021; Lin et al., 2021) were used to verify FDR results and explore the additional potential differences between the two groups.

### Correlations With Clinical Variables in Migraine

Two sample *T*-test was used to interrogate demographic differences between MWoAs and healthy controls. Through the normal distribution test, it was found that the clinical data did not conform to the normal distribution, so Spearman correlation was used to analyze the clinical correlations of the MWoA group in GM-averaged CC of each bundle and WM-averaged CC of each GM region. Thresholds were *p* < 0.05, and FDR was corrected for multiple comparisons.

## Results

### Demographic and Clinical Characteristics

The demographic and clinical data of the migraine group and control group are shown in Table 1. There was no difference in age and gender between the two groups.

### Resting-State Correlations Between White Matter and Gray Matter

We applied a false positive correction of FDR to determine the significance level between the two groups. And *p* < 1/N correction was used to verify and compare the results. There were 82 GM regions and 48 WM bundles; when analyzing GM-averaged correlation coefficients of WM bundles, N is 48, *P* < 0.020833 (Table 2); while when analyzing WM averaged correlation coefficients of GM bundles, N is 82, *P* < 0. 0.012195 (Table 3). Comparing *P* < 1/N and FDR correction, we found that *P* < 1/N correction had more positive results when *N* = 48, and the results were more similar when *N* = 82. For *P* < 1/N correction, the larger the N, the greater the correction effect.

**TABLE 2:** Summary of *t*-tests of GM-averaged correlation coefficients of WM bundles in migraine without aura (MWoA) compared to healthy controls (HC).

Bundle	Right						Left					
	HC		MWoA				HC		MWoA			
	Mean	*SD*	Mean	*SD*	*P*	*P* (FDR)	Mean	*SD*	Mean	*SD*	*P*	*P* (FDR)
Corticospinal tract	0.358	0.151	0.310	0.151	0.137	0.193	0.349	0.157	0.293	0.152	0.081	0.134
Medial lemniscus	0.194	0.149	0.12	0.150	**0.016** *	0.081	0.192	0.161	0.136	0.137	0.063	0.125
Inferior cerebellar peduncle	0.282	0.14	0.209	0.133	**0.009** *	0.081	0.267	0.136	0.238	0.138	0.300	0.351
Superior cerebellar peduncle	0.224	0.135	0.152	0.145	**0.013** *	0.081	0.246	0.149	0.172	0.146	**0.018** *	0.081
Cerebral peduncle	0.366	0.127	0.299	0.154	0.031	0.096	0.351	0.136	0.314	0.141	0.213	0.269
Anterior limb of internal capsule	0.385	0.143	0.325	0.145	0.042	0.096	0.374	0.145	0.325	0.151	0.086	0.137
Posterior limb of internal capsule	0.398	0.138	0.340	0.154	0.047	0.103	0.389	0.141	0.338	0.149	0.079	0.134
Retrolenticular part of internal capsule	0.306	0.142	0.263	0.126	0.093	0.137	0.276	0.153	0.226	0.122	0.065	0.125
Anterior corona radiata	0.246	0.134	0.177	0.131	0.013*	0.081	0.248	0.125	0.163	0.129	**0.002** *	**0.048** ^#^
Superior corona radiata	0.235	0.146	0.167	0.145	0.022	0.081	0.227	0.141	0.169	0.146	0.051	0.106
Posterior corona radiata	0.148	0.148	0.096	0.137	0.073	0.130	0.157	0.148	0.096	0.143	0.04	0.096
Posterior thalamic radiation (Include OR)	0.15	0.152	0.102	0.134	0.094	0.137	0.195	0.135	0.132	0.125	0.020*	0.081
Sagittal stratum	0.247	0.124	0.216	0.12	0.204	0.265	0.246	0.129	0.198	0.121	0.069	0.127
External capsule	0.474	0.127	0.415	0.122	0.022	0.081	0.501	0.125	0.424	0.122	**0.003** *	**0.048** ^#^
Cingulum (Cingulate gyrus)	0.461	0.13	0.403	0.135	0.037	0.096	0.450	0.125	0.389	0.129	0.024	0.082
Cingulum (Hippocampus)	0.36	0.135	0.357	0.12	0.947	0.947	0.387	0.145	0.345	0.134	0.176	0.235
Fornix (Cres)/Stria terminalis	0.339	0.149	0.321	0.127	0.468	0.488	0.336	0.143	0.318	0.124	0.459	0.488
Superior longitudinal fasciculus	0.293	0.14	0.209	0.129	**0.004** *	**0.048** ^#^	0.311	0.132	0.233	0.125	**0.004** *	**0.048** ^#^
Superior fronto-occipital fasciculus	0.267	0.154	0.226	0.142	0.159	0.218	0.269	0.141	0.224	0.13	0.093	0.137
Uncinate fasciculus	0.332	0.133	0.311	0.127	0.453	0.488	0.349	0.143	0.297	0.105	0.04	0.096
Tapetum	0.02	0.181	0.003	0.125	0.538	0.549	0.061	0.168	0.028	0.142	0.282	0.338

**Bundle**	**HC**					**MWoA**						
	**Mean**		**SD**			**Mean**		**SD**		**P**		**P (FDR)**

Middle cerebellar peduncle	0.345		0.156			0.268		0.158		**0.015** *		0.081
Pontine crossing tract (Part of MCP)	0.325		0.167			0.258		0.15		0.039		0.096
Genu of corpus callosum	0.318		0.137			0.260		0.129		0.032		0.096
Body of corpus callosum	0.152		0.161			0.118		0.153		0.275		0.338
Splenium of corpus callosum	0.278		0.142			0.252		0.140		0.394		0.450
Fornix (Column and body of fornix)	0.261		0.160			0.234		0.164		0.405		0.452

The P-values are based on two-tailed t-tests of Z scores of the correlation coefficients. The results were assigned thresholds at p < 0.05 (FDR adjusted). The asterisks (*) denote p < 1/N(0.020833). The (^#^) denote p (FDR adjusted) < 0.05. The bold values represented significant results after indicated FDR correction or p < 1/N correction.

**TABLE 3:** Summary of *t*-tests of WM-averaged correlation coefficients of GM bundles in migraine without aura (MWoA) compared to healthy controls (HC).

Brodmann ROI		Right						Left					
		HC		MWoA				HC		MWoA			
		Mean	*SD*	Mean	*SD*	*P*	*P* (FDR)	Mean	*SD*	Mean	*SD*	*P*	*P* (FDR)
BA1	Primary somatosensory cortex1	0.193	0.138	0.175	0.144	0.466	0.484	0.185	0.144	0.126	0.135	0.035	0.074
BA2	Primary somatosensory cortex2	0.239	0.143	0.177	0.161	0.040	0.076	0.254	0.125	0.170	0.140	**0.003** *	**0.038** ^#^
BA3	Primary somatosensory cortex3	0.235	0.156	0.184	0.178	0.110	0.139	0.229	0.148	0.164	0.173	0.046	0.077
BA4	Primary motor cortex	0.251	0.158	0.219	0.163	0.291	0.310	0.244	0.161	0.213	0.159	0.284	0.306
BA5	Somatosensory association cortex	0.328	0.133	0.258	0.125	**0.008** *	**0.041** ^#^	0.315	0.133	0.246	0.119	**0.006** *	**0.038** ^#^
BA6	Premotor and supplementary motor cortex	0.306	0.156	0.251	0.165	0.079	0.112	0.308	0.148	0.255	0.159	0.088	0.122
BA7	Visuo-motor coordination	0.325	0.133	0.246	0.136	**0.003** *	**0.038** ^#^	0.313	0.124	0.236	0.143	**0.003** *	**0.038** ^#^
BA8	Includes frontal eye fields	0.280	0.141	0.234	0.100	0.039	0.076	0.304	0.129	0.229	0.108	**0.002** *	**0.038**
BA9	Dorsolateral prefrontal cortex	0.299	0.134	0.251	0.097	0.025	0.062	0.305	0.134	0.244	0.106	**0.010** *	**0.043** ^#^
BA10	Anterior prefrontal cortex	0.335	0.109	0.273	0.104	**0.004** *	**0.038** ^#^	0.331	0.113	0.266	0.114	**0.006** *	**0.038** ^#^
BA11	Orbitofrontal area	0.257	0.117	0.234	0.112	0.229	0.254	0.262	0.126	0.237	0.117	0.224	0.254
BA17	Primary visual cortex (V1)	0.296	0.145	0.233	0.133	0.024	0.062	0.286	0.140	0.226	0.137	0.036	0.074
BA18	Secondary visual cortex (V2)	0.360	0.151	0.296	0.127	0.019	0.056	0.367	0.145	0.319	0.114	0.047	0.077
BA19	Associative visual cortex (V3, V4, V5)	0.378	0.139	0.319	0.132	0.022	0.060	0.356	0.141	0.309	0.123	0.051	0.077
BA20	Inferior temporal gyrus	0.332	0.126	0.284	0.137	0.051	0.077	0.335	0.131	0.293	0.136	0.097	0.124
BA21	Middle temporal gyrus	0.282	0.132	0.223	0.114	0.013	**0.049** ^#^	0.292	0.138	0.239	0.115	0.029	0.066
BA22	Superior temporal gyrus	0.267	0.160	0.202	0.126	0.020	0.057	0.261	0.150	0.210	0.123	0.047	0.077
BA23	Ventral posterior cingulate cortex	0.35	0.123	0.296	0.095	0.016	0.051	0.351	0.119	0.294	0.096	**0.009** *	**0.043** ^#^
BA24	Ventral anterior cingulate cortex	0.367	0.130	0.310	0.113	0.019	0.056	0.367	0.129	0.300	0.113	**0.008** *	**0.041** ^#^
BA25	Subgenual area	0.274	0.116	0.233	0.118	0.07	0.103	0.261	0.110	0.237	0.127	0.299	0.314
BA26	Ectosplenial portion of the retrosplenial region	0.325	0.125	0.257	0.128	**0.006** *	**0.038** ^#^	0.329	0.117	0.263	0.116	**0.005** *	**0.038** ^#^
BA27	Piriform cortex	0.352	0.136	0.294	0.122	0.029	0.066	0.318	0.128	0.266	0.108	0.03	0.067
BA28	Ventral entorhinal cortex	0.148	0.137	0.137	0.140	0.664	0.672	0.148	0.101	0.139	0.133	0.677	0.677
BA29	Retrosplenial cingulate cortex	0.29	0.125	0.257	0.098	0.127	0.153	0.304	0.123	0.269	0.102	0.125	0.153
BA30	Part of cingulate cortex	0.367	0.133	0.317	0.110	0.039	0.076	0.361	0.132	0.307	0.118	0.041	0.076
BA32	Dorsal anterior cingulate cortex	0.347	0.129	0.298	0.103	0.026	0.063	0.354	0.129	0.291	0.111	**0.010** *	**0.043** ^#^
BA34	Dorsal entorhinal cortex (Parahippocampal gyrus)	0.295	0.137	0.267	0.118	0.229	0.254	0.257	0.134	0.241	0.121	0.484	0.496
BA35	Perirhinal cortex	0.239	0.145	0.200	0.131	0.155	0.182	0.225	0.129	0.195	0.128	0.247	0.270
BA36	Ectorhinal area, now part of the perirhinal cortex	0.249	0.135	0.206	0.135	0.093	0.123	0.259	0.142	0.220	0.134	0.127	0.153
BA37	Fusiform gyrus	0.404	0.135	0.361	0.124	0.076	0.109	0.385	0.139	0.345	0.130	0.089	0.122
BA38	Temporopolar area	0.268	0.128	0.220	0.120	0.046	0.077	0.249	0.123	0.210	0.116	0.093	0.123
BA39	Angular gyrus, may be part of wernicke	0.273	0.130	0.218	0.099	0.014	0.050	0.267	0.120	0.219	0.104	0.035	0.074
BA40	Supramarginal gyrus, may be part of wernicke	0.264	0.147	0.199	0.133	0.016	0.051	0.261	0.130	0.184	0.124	**0.003** *	**0.038** ^#^
BA41	Auditory cortex1	0.272	0.140	0.224	0.147	0.096	0.124	0.260	0.130	0.206	0.102	0.016	0.051
BA42	Auditory cortex2	0.267	0.152	0.194	0.131	**0.011** *	**0.045** ^#^	0.259	0.143	0.209	0.120	0.047	0.077
BA43	Primary gustatory cortex	0.155	0.153	0.113	0.145	0.139	0.165	0.165	0.158	0.127	0.145	0.179	0.207
BA44	Pars opercularis, part of IFG and part of broca	0.230	0.148	0.179	0.133	0.051	0.077	0.249	0.139	0.178	0.139	**0.012** *	**0.047** ^#^
BA45	Pars triangularis, part of IFG and part of broca	0.265	0.155	0.215	0.115	0.042	0.077	0.287	0.137	0.216	0.118	**0.006** *	**0.038** ^#^
BA46	Dorsolateral prefrontal cortex	0.360	0.144	0.290	0.110	**0.004** *	**0.038** ^#^	0.341	0.148	0.275	0.117	**0.007** *	**0.041** ^#^
BA47	Pars orbitalis, part of the inferior frontal gyrus	0.312	0.139	0.247	0.101	**0.005** *	**0.038** ^#^	0.297	0.132	0.246	0.110	0.025	0.062
BA48	Retrosubicular area	0.340	0.143	0.288	0.126	0.051	0.077	0.341	0.136	0.292	0.121	0.066	0.098

The P-values are based on two-tailed t-tests of Z scores of the correlation coefficients. The results were assigned thresholds at p < 0.05 (FDR adjusted). The asterisks (*) denote p < N/1. The (^#^) denote p (FDR adjusted) < 0.05. The bold values represented significant results after indicated FDR correction or p < 1/N correction.

**FIGURE 2:**
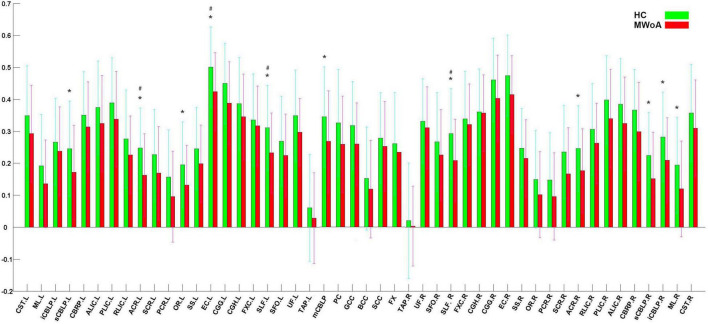
Quantitative comparisons between patients with MWoA and HCs in GM-averaged correlation coefficients of each bundle. The asterisks (*) denote *p* < N/1(0. 0.012195). The (^#^) denote *p* (FDR adjusted) < 0.05.

**FIGURE 3:**
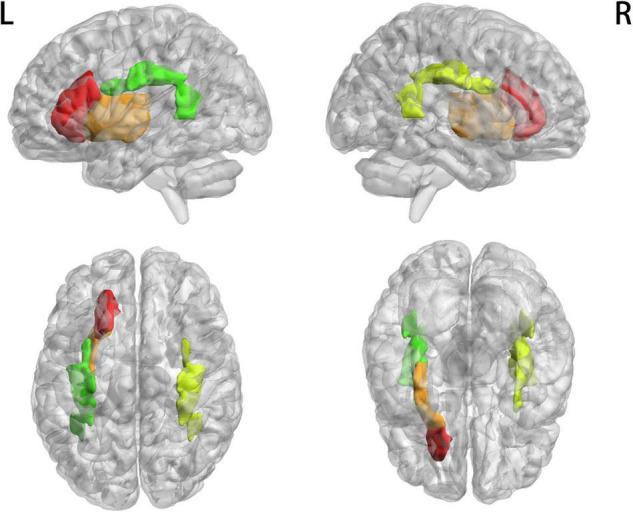
WM bundles in the cerebrum of MNI space with decreased GM-averaged correlation coefficients. Thresholds were *p* < 0.05, and FDR corrected for multiple comparisons.

Figure 1 shows that most of the functional connections between the GM regions and the WM fibers were reduced in patients with MWoA relative to those in HC, while little was increased. Quantitative comparisons between MWoA and HC in the mean CC of the GM regions across the WM bundles in the resting state are given in Figure 2 and Table 2. After FDR correction, left anterior corona radiata, left external capsule and bilateral superior longitudinal fasciculus had significantly reduced mean CCs with GM in MWoA. The WM bundles in the cerebrum of the MNI space with decreased GM-averaged CC after FDR correction are shown in Figure 3. While *p* < 1/N correction showed that other 5 WM bundles had significantly decreased mean CCs with GM in patients with MWoA, including some tracts in the brainstem such as the right medial lemniscus, the right inferior cerebellar peduncle (ICP), the middle cerebellar peduncle (MCP), and the bilateral superior cerebellar peduncle (SCP), and some projection fibers such as the bilateral anterior corona radiate. In addition, association fibers such as the left posterior thalamic radiation (include optic radiation) were also affected.

Quantitative comparisons between MWoA and HC for the mean CC of the GM bundles across the WM regions at the resting state are given in Supplementary Figure 2 and Table 3. After FDR correction, they show that 16 GM regions had significantly decreased mean CCs with WM in patients with MWoA, such as sensorimotor regions including the left primary somatosensory cortex, the bilateral somatosensory association cortex, the bilateral visuomotor coordination, and the auditory cortex including the right auditory cortex, and frontal areas such as the bilateral anterior prefrontal cortex, the bilateral dorsolateral prefrontal cortex, the bilateral pars orbitalis, the left frontal eye fields, the left dorsolateral prefrontal cortex, the left pars triangularis, and the right middle temporal gyrus, and the cingulate cortex including the left ventral posterior cingulate cortex, the left ventral anterior cingulate cortex, the bilateral ectosplenial portion of the retrosplenial region, the left dorsal anterior cingulate cortex, and the left supramarginal gyrus. *P* < 1/N correction showed similar results with FDR, and only the right middle temporal gyrus pass FDR correction but did not pass *P* < 1/N correction. The GM regions in the cerebrum of the MNI space with decreased WM-averaged CCs are shown in Supplementary Figure 3.

### Correlations With Clinical Variables in Migraine

**FIGURE 4:**
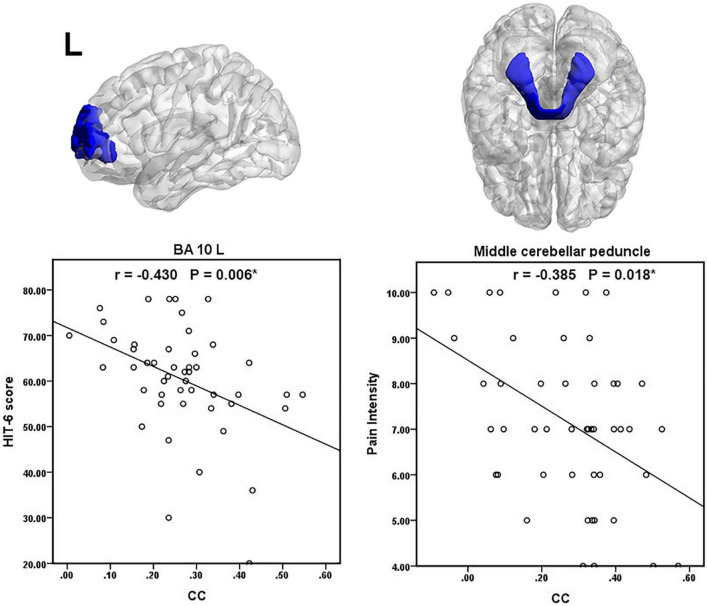
Scatter plot of significant clinical correlations. The (*) denote *p* (FDR adjusted) < 0.05.

We found a negative correlation between the patients’ HIT- 6 score and the WM-averaged CC in the left anterior prefrontal cortex. The patient’s pain intensity during headache attacks was negatively correlated with the GM-averaged CC in the middle cerebellar peduncle. A scatter plot of significant clinical relevance is shown in Figure 4. Detailed clinical correlation coefficients are given in Supplementary Tables 2, 3.

## Discussion

In the present study, we observed the functional connection between WM bundles and GM regions in MWoAs, which made up the gap between the previous studies of WM and GM. Patients with MWoAs exhibited overall decreased average temporal CCs between BOLD signals in 48 WM bundles and 82 GM regions compared with HCs, while little was increased. In particular, after FDR correction left anterior corona radiata, left external capsule and bilateral superior longitudinal fasciculus had significantly decreased mean CCs with the GM in patients with MWoA. While *p* < 1/N correction showed that other 5 WM bundles had significantly decreased mean CCs with GM in patients with MWoA. These results are also discussed as exploratory results, revealing underlying WM functional change. The WM fibers had decreased mean CCs with the GM in patients with MWoA, revealing functional alterations of the WM fibers. The functional alterations in the WM fibers might lead to a disruption in the transmission of signals between the nerve centers, eventually leading to dysfunction in the functional information integration between migraine-relevant centers.

### Projection Fibers

Left anterior corona radiata had significantly decreased mean CC with GM, after FDR correction. Afferent and efferent projection fibers provide the connection between the cerebral cortex, brainstem, and spinal cord (Yakar et al., 2018). All of these fibers are distributed together as fan-shaped structures in a hemisphere and form the corona radiata (Yakar et al., 2018). The corona radiata is divided into three regions: anterior corona radiata, superior corona radiata, and posterior corona radiata. This region includes the thalamic radiations (corticothalamic, thalamocortical fibers) and parts of the long corticofugal pathways, such as the corticopontine tracts, corticospinal tracts, and corticobulbar tracts. Previous studies have shown that migraine patients exhibited decreased FA of the corona radiata at the level of the lateral ventricle horn along the trigeminothalamic tract (DaSilva et al., 2007). Patients’ decreased functional connections in the anterior corona radiata with GM regions might lead to deficits in functional information transfer between the cerebral cortex, brainstem, and spinal cord.

### Association Fibers

After FDR correction, the bilateral SLF and left external capsule exhibited reduced mean CC with GM. The SLF is located in the dorsolateral regions of the corona radiata and connects the front and back of the cerebrum, including the frontal, parietal, occipital, and temporal lobes (Leung et al., 2018). SLF deficits in connecting these brain regions can affect the effectiveness of the prefrontal cortex in modulating the abovementioned multiple aspects of sensory or pain perception and can eventually lead to the development of headaches. The external capsule is located lateral to the internal capsule and is thought to contain association fibers, such as the SLF and inferior fronto-occipital fasciculus and commissural fibers (Mori et al., 2008). The extreme and external capsules lie in an anteroposterior disposition and are connected with the insula’s anteroinferior part (Ribas et al., 2018). The lentiform nucleus is located between the internal and external capsules (Ribas et al., 2018). The external, internal, and extreme capsules connect the insular surface, basal ganglia, and thalamus to the cerebral lobes (Ribas et al., 2018). Thus, the external capsule is involved in anteroposterior connections and connections between subcortical and cortical regions relevant to migraine pathogenesis. Our results showed left external capsule decreased functional connectivity with GM in patients with MWoA, which might lead to impaired functional integration between numerous brain regions and may be related to patients’ recurrent headaches and multiple sensory symptoms.

After *P* < 1/N correction, we found that patients with MWoA exhibited decreased GM-averaged CCs of the left posterior thalamic radiation (including optic radiation) and that this functional alteration might contribute to deficits in thalamic relay and modulation of the motor, sensory, and pain information, as well as dysfunction in visual information processing. Posterior thalamic radiation includes the corticothalamic and thalamocortical fibers and contains optic radiation (Mori et al., 2008). Thalamocortical activity plays an important role in abnormal sensory processing, which is a central feature of migraine attacks and could be used as a therapeutic target for pharmacological and neuromodulatory methods (such as transcranial magnetic stimulation) (Andreou et al., 2016). The thalamus’s primary function is to transmit and regulate motor and sensory information, including pain modulation between numerous cortical regions and the peripheral nervous system (Herrero et al., 2002). Our previous study found that patients with MWoA exhibited a diminished level of functional connectivity between the subregions of the thalamus and left precuneus, right inferior parietal lobule, and right middle frontal gyrus (Qin et al., 2020c). A visual task specifically induced a stronger degree of temporal coherence within the optic radiations compared with the resting state, as well as significant correlations between the optic radiations and multiple cortical visual networks (Marussich et al., 2017); thus, optic radiation provides rich visual processing information. Although the migraine patients in this study had no aura, they had no visual aura; we observed WM abnormalities in the visual area. It is controversial whether there is an essential difference between migraine with aura and migraine without aura (Vgontzas and Burch, 2018). Migraine patients (both with and without aura) may be related to the abnormal integration of somatosensory, visual, auditory, and olfactory stimuli (Schwedt, 2013). Migraine patients with aura are explicitly related to visual hypersensitivity (Pearl et al., 2020). We speculate migraine with and without aura both have abnormal integration of sensory information and pain processing. Migraine with aura may be more sensitive to some kind of sensory sensitivity, such as visual sensitivity, and have a visual aura. In contrast, those who are not sensitive to visuals may have a “silent aura.”

### Tracts in the Brainstem

After *p* < 1/N correction, we found that right ICP, MCP, and bilateral SCP had decreased mean CCs with the GM in MWoA. The cerebellar peduncles are major white matter tracts of the cerebellum that communicate information among the cerebral cortex, the spinal cord, and the cerebellum. The ICPs connect the spinal cord, medulla, and cerebellum, carrying a variety of input and output fibers, which is mainly related to the integration of proprioception sensory input with motor vestibular function (Mori et al., 2005).

The MCP is emitted from pontine nuclei and transmits information between the cortex and cerebellum (Mori et al., 2008). The SCP transmits information between the dentate nucleus and thalamus (Mori et al., 2008). Migraine patients have a lower SCP volume and migraineurs with lower heat pain thresholds have smaller SCPs (Chong et al., 2017). Our previous study investigated that MWoAs exhibited altered microstructure in the ICP tract (Qin et al., 2019). These WM functional alterations in cerebellar peduncle tracts may cut off the relay stations in the cerebrocerebellar circuitry, eventually leading to dysfunction of conduction and integration of sensory and motor information among the medulla, the cerebellum, and cerebral cortex. The pain intensity of the migraineurs was negatively correlated with the GM-averaged CC in the middle cerebellar peduncle. This result further indicates that the reduced GM-averaged CC in the middle cerebellar peduncle is closely related to pain intensity in MWoAs.

After *P* < 1/N correction, our results showed that patients with MWoA exhibited decreased GM-averaged CCs at the right medial lemniscus, and this functional abnormality might contribute to patients with MWoA suffering from altered sensory information processing. The medial lemniscus travels along the dorsal side of the midbrain and pons and turns sharply toward the brainstem’s ventral side at the level of the medulla (Wakana et al., 2004). The medial lemniscus is the primary sensory pathway toward the thalamus (Mori et al., 2008). Diffusion-weighted images analysis showed that fractional anisotropy (FA) in the region of the medial lemniscus/ventral trigeminal thalamic tract was significantly elevated in migraineurs compared with controls over the entire migraine cycle (Marciszewski et al., 2019).

### Gray Matter Regions

On the other hand, 16 GM regions had significantly decreased mean CCs with WM in patients with MWoA (16 GM regions after FDR correction, 15 GM regions after *P* < 1/N correction), including some areas that are part of the somatosensory regions, auditory cortex, frontal areas, temporal gyrus, cingulate cortex, and parietal cortex. In line with previous studies (Zhang et al., 2016, 2017a,b; Jia and Yu, 2017; Ashina et al., 2021; Kim et al., 2021), migraine as a complex brain disease involves functional abnormalities in multiple cortical regions. These GM regions are believed to play critical roles in the pathophysiology of migraines. Our previous study indicated disrupted functional connectivity between the thalamus, sensorimotor areas, posterior pons, and other migraine-relevant brain regions in MWoA patients (Qin et al., 2020a,b,c). We speculated that the disrupted functional connections of the cortical regions in migraine patients may be due to the abnormal functional coupling between GM regions and WM regions. For example, the sensorimotor cortex’s altered interactions with WM areas might contribute to the sensorimotor functional abnormalities and disrupted FC. Thus, our results further suggest that functional abnormalities and connectivity abnormalities in cortical regions may not be limited to gray matter regions but may be associated with functional abnormalities in white matter fiber tracts. In addition, correlation analysis, we obtained significantly negative correlations between HIT-6 scores and WM-averaged CCs in the left anterior prefrontal cortex, which suggested that the decrease of CC is significantly related to the impact of recurrent migraine attacks on the quality of daily life.

### Limitation

The current study has several limitations. Firstly, only 82 GM regions of the cortex were considered, while subcortical regions were not included in the analysis. The structure of the subcortical brain area is more complex, including neurons and a large number of nerve fibers. The functional connections between subcortical areas and WM bundles also need to be further studied. In addition, we found no significant difference between male patients and female patients, and gender was used as a covariate in the analysis. In this study, women are the majority; the gender differences need to be further investigated in the future.

## Conclusion

The patients with MWoA showed overall disrupted functional connections between WM bundles and GM regions compared with HCs, which might contribute to disrupted functional connectivity of the pain processing pathway. WM fibers exhibited functional abnormalities in some tracts in the brainstem and in some projection and association fibers in patients with MWoA, which might lead to dysfunctions in the conduction and integration of multisensory and motor information between cortical regions relevant in migraine pathogenesis and to deficits in the modulation of multiple aspects of sensory or pain perception, as well as abnormalities in visual information processing. Moreover, GM regions that play critical roles in migraine pathology exhibited decreased functional connectivity with WM areas in patients with MWoA. Thus, in patients with MWoA, functional abnormalities and connectivity abnormalities in cortical regions may not be limited to gray matter regions and are associated with functional abnormalities in white matter fiber tracts. Pain sensitivity and patient quality of life are closely tied to abnormal functional interactions between WM and GM areas. These findings provide new insights into the neural basis of migraine pathology and will benefit the diagnosis and treatment of this disorder.

## Data Availability Statement

The raw data supporting the conclusions of this article will be made available by the authors, without undue reservation.

## Ethics Statement

The studies involving human participants were reviewed and approved by the Independent Ethics Committee of Shanghai Ninth People’s Hospital [Project No. (2016) 01] and the Human Research Committee of East China Normal University (Project No. HR2015/03011). The patients/participants provided their written informed consent to participate in this study.

## Author Contributions

ZQ analyzed the data and prepared the manuscript. H-BL was responsible for evaluating the clinical data of the subjects and conducting experiments. ML was responsible for guiding the analysis of fMRI data. YH assisted in the collection of clinical data and the implementation of experiments. JW and YQ participated in the collection of clinical data and the implementation of experiments. J-RL designed the study and reviewed the manuscript. XD designed the study and prepared, revised the manuscript. All authors read and approved the final manuscript.

## Conflict of Interest

The authors declare that the research was conducted in the absence of any commercial or financial relationships that could be construed as a potential conflict of interest.

## Publisher’s Note

All claims expressed in this article are solely those of the authors and do not necessarily represent those of their affiliated organizations, or those of the publisher, the editors and the reviewers. Any product that may be evaluated in this article, or claim that may be made by its manufacturer, is not guaranteed or endorsed by the publisher.

## Footnotes

1
http://rfmri.org/DPARSF

2
http://www.fil.ion.ucl.ac.uk/spm
