# Supplementary material for: Disrupted White Matter Functional Connectivity With the Cerebral Cortex in Migraine Patients
Source: Front Neurosci. 2022 Jan 13;15:799854. doi: 10.3389/fnins.2021.799854 (PMC8793828; doi:10.3389/fnins.2021.799854)
Supplement: Supplementary file 1 [file Data_Sheet_1.docx]

**Stable I.** List of abbreviations for WM bundles

| Abbreviation | Full name |
| --- | --- |
| CST | Corticospinal tract |
| ML | Medial lemniscus |
| iCBLP | Inferior cerebellar peduncle |
| sCBLP | Superior cerebellar peduncle |
| CBRP | Cerebral peduncle |
| ALIC | Anterior limb of internal capsule |
| PLIC | Posterior limb of internal capsule |
| RLIC | Retrolenticular part of internal capsule |
| ACR | Anterior corona radiata |
| SCR | Superior corona radiata |
| PCR | Posterior corona radiata |
| OR | optic radiation |
| SS | Sagittal stratum |
| EC | External capsule |
| CGG | Cingulum (cingulate gyrus) |
| CGH | Cingulum (hippocampus) |
| FXC | Fornix (cres) / Stria terminalis |
| SLF | Superior longitudinal fasciculus |
| SFO | Superior fronto-occipital fasciculus |
| UF | Uncinate fasciculus |
| TAP | Tapetum |
| mCBLP | Middle cerebellar peduncle |
| PC | Pontine crossing tract (part of MCP) |
| GCC | Genu of corpus callosum |
| BCC | Body of corpus callosum |
| SCC | Splenium of corpus callosum |
| FX | Fornix (column and body of fornix) |

**Stable Ⅱ.** Clinical correlations with decreased WM-averaged correlation coefficients of GM regions in patients with MWoA.

| Brodmann ROI |  | Disease duration (years) | Duration (hours) | Frequency (times/months) | Pain intensity (VAS) | MIDAS | HIT-6 |
| --- | --- | --- | --- | --- | --- | --- | --- |
| BA5.R | r | 0.121 | 0.030 | -0.071 | -0.307 | -0.129 | -0.292 |
|  | p value | 0.309 | 0.419 | 0.380 | 0.066 | 0.309 | 0.066 |
| BA7.R | r | -0.109 | 0.004 | -0.111 | -0.238 | -0.053 | -0.125 |
|  | p value | 0.345 | 0.490 | 0.345 | 0.312 | 0.431 | 0.345 |
| BA10.R | r | -0.079 | -0.101 | 0.005 | -0.202 | 0.021 | -0.298 |
|  | p value | 0.446 | 0.446 | 0.485 | 0.252 | 0.485 | 0.120 |
| BA26.R | r | 0.084 | 0.155 | -0.049 | -0.083 | -0.155 | -0.311 |
|  | p value | 0.343 | 0.294 | 0.371 | 0.343 | 0.294 | 0.096 |
| BA42.R | r | -0.035 | -0.077 | -0.036 | -0.222 | 0.080 | -0.269 |
|  | p value | 0.407 | 0.407 | 0.407 | 0.195 | 0.407 | 0.192 |
| BA46.R | r | 0.008 | -0.153 | -0.031 | -0.130 | 0.134 | -0.202 |
|  | p value | 0.478 | 0.284 | 0.478 | 0.284 | 0.284 | 0.284 |
| BA47.R | r | 0.051 | 0.007 | -0.075 | -0.135 | -0.087 | -0.246 |
|  | p value | 0.438 | 0.482 | 0.438 | 0.438 | 0.438 | 0.276 |
| BA46.L | r | -0.003 | -0.096 | -0.117 | -0.222 | 0.144 | -0.254 |
|  | p value | 0.492 | 0.310 | 0.310 | 0.192 | 0.310 | 0.192 |
| BA45.L | r | 0.309 | 0.082 | -0.275 | -0.070 | -0.035 | -0.210 |
|  | p value | 0.087 | 0.382 | 0.087 | 0.382 | 0.406 | 0.152 |
| BA40.L | r | -0.003 | 0.063 | -0.296 | -0.174 | 0.062 | -0.135 |
|  | p value | 0.491 | 0.406 | 0.126 | 0.357 | 0.406 | 0.362 |
| BA32.L | r | 0.114 | -0.229 | 0.045 | -0.091 | 0.082 | -0.273 |
|  | p value | 0.348 | 0.177 | 0.381 | 0.348 | 0.348 | 0.177 |
| BA26.L | r | 0.199 | 0.115 | -0.028 | -0.126 | -0.143 | -0.316 |
|  | p value | 0.262 | 0.262 | 0.425 | 0.262 | 0.262 | 0.084 |
| BA24.L | r | 0.186 | -0.222 | 0.021 | 0.000 | 0.127 | -0.276 |
|  | p value | 0.204 | 0.195 | 0.500 | 0.500 | 0.293 | 0.174 |
| BA23.L | r | 0.128 | 0.125 | -0.082 | -0.207 | -0.145 | -0.329 |
|  | p value | 0.239 | 0.239 | 0.290 | 0.237 | 0.239 | 0.066 |
| BA10.L | r | -0.120 | -0.108 | 0.085 | -0.211 | -0.002 | **-0.430** |
|  | p value | 0.340 | 0.340 | 0.340 | 0.225 | 0.494 | **0.006*** |
| BA9.L | r | 0.131 | -0.008 | -0.066 | -0.255 | 0.041 | -0.219 |
|  | p value | 0.376 | 0.480 | 0.468 | 0.204 | 0.468 | 0.204 |
| BA8.L | r | 0.115 | 0.132 | -0.158 | -0.090 | -0.102 | -0.235 |
|  | p value | 0.272 | 0.272 | 0.272 | 0.272 | 0.272 | 0.272 |
| BA7.L | r | -0.069 | 0.018 | -0.199 | -0.290 | -0.068 | -0.194 |
|  | p value | 0.388 | 0.452 | 0.186 | 0.138 | 0.388 | 0.186 |
| BA5.L | r | 0.113 | -0.002 | -0.121 | -0.318 | -0.072 | -0.249 |
|  | p value | 0.335 | 0.496 | 0.335 | 0.084 | 0.377 | 0.132 |
| BA2.L | r | 0.021 | 0.098 | -0.269 | -0.229 | -0.069 | -0.128 |
|  | p value | 0.443 | 0.381 | 0.177 | 0.177 | 0.386 | 0.381 |

P value, Sig. (FDR corrected); r, Spearson’s rho. *Statistical significance at FDR-corrected P <0.05.

**Stable Ⅲ.** Clinical correlations with decreased GM-averaged correlation coefficients of WM bundles in patients with MWoA.

| WM bundles |  | Disease duration (years) | Duration (hours) | Frequency (times/months) | Pain intensity (VAS) | MIDAS | HIT-6 |
| --- | --- | --- | --- | --- | --- | --- | --- |
| Medial lemniscus R | r | 0.000 | 0.038 | -0.122 | -0.143 | -0.087 | 0.015 |
|  | p value | 0.499 | 0.499 | 0.499 | 0.499 | 0.499 | 0.499 |
| Inferior cerebellar peduncle R | r | -0.149 | -0.173 | -0.100 | -0.248 | -0.021 | -0.272 |
|  | p value | 0.234 | 0.234 | 0.299 | 0.135 | 0.445 | 0.135 |
| Superior cerebellar peduncle R | r | -0.063 | -0.122 | -0.143 | -0.188 | 0.059 | -0.150 |
|  | p value | 0.344 | 0.308 | 0.308 | 0.308 | 0.344 | 0.308 |
| Anterior corona radiata R | r | 0.073 | 0.151 | -0.010 | -0.237 | -0.070 | -0.100 |
|  | p value | 0.382 | 0.382 | 0.472 | 0.312 | 0.382 | 0.382 |
| Superior longitudinal fasciculus R | r | 0.250 | 0.199 | -0.124 | -0.039 | -0.174 | -0.137 |
|  | p value | 0.236 | 0.236 | 0.241 | 0.396 | 0.236 | 0.241 |
| Middle cerebellar peduncle | r | -0.084 | -0.174 | -0.052 | **-0.385** | 0.051 | -0.154 |
|  | p value | 0.366 | 0.298 | 0.366 | **0.018*** | 0.366 | 0.298 |
| Superior longitudinal fasciculus L | r | 0.226 | 0.165 | -0.118 | -0.152 | -0.134 | -0.291 |
|  | p value | 0.183 | 0.213 | 0.213 | 0.213 | 0.213 | 0.138 |
| External capsule L | r | 0.121 | -0.117 | -0.069 | -0.060 | 0.043 | -0.317 |
|  | p value | 0.386 | 0.386 | 0.386 | 0.386 | 0.386 | 0.084 |
| Posterior thalamic radiation (include optic radiation) L | r | 0.210 | 0.179 | -0.202 | -0.201 | -0.280 | -0.167 |
|  | p value | 0.128 | 0.128 | 0.128 | 0.128 | 0.128 | 0.128 |
| Anterior corona radiata L | r | 0.132 | 0.132 | -0.101 | -0.246 | -0.030 | -0.027 |
|  | p value | 0.370 | 0.370 | 0.372 | 0.276 | 0.429 | 0.429 |
| Superior cerebellar peduncle L | r | -0.073 | -0.136 | -0.162 | -0.232 | 0.185 | -0.144 |
|  | p value | 0.312 | 0.215 | 0.215 | 0.215 | 0.215 | 0.215 |

P value, Sig. (FDR corrected); r, Spearson’s rho. *Statistical significance at FDR-corrected P < 0.05.


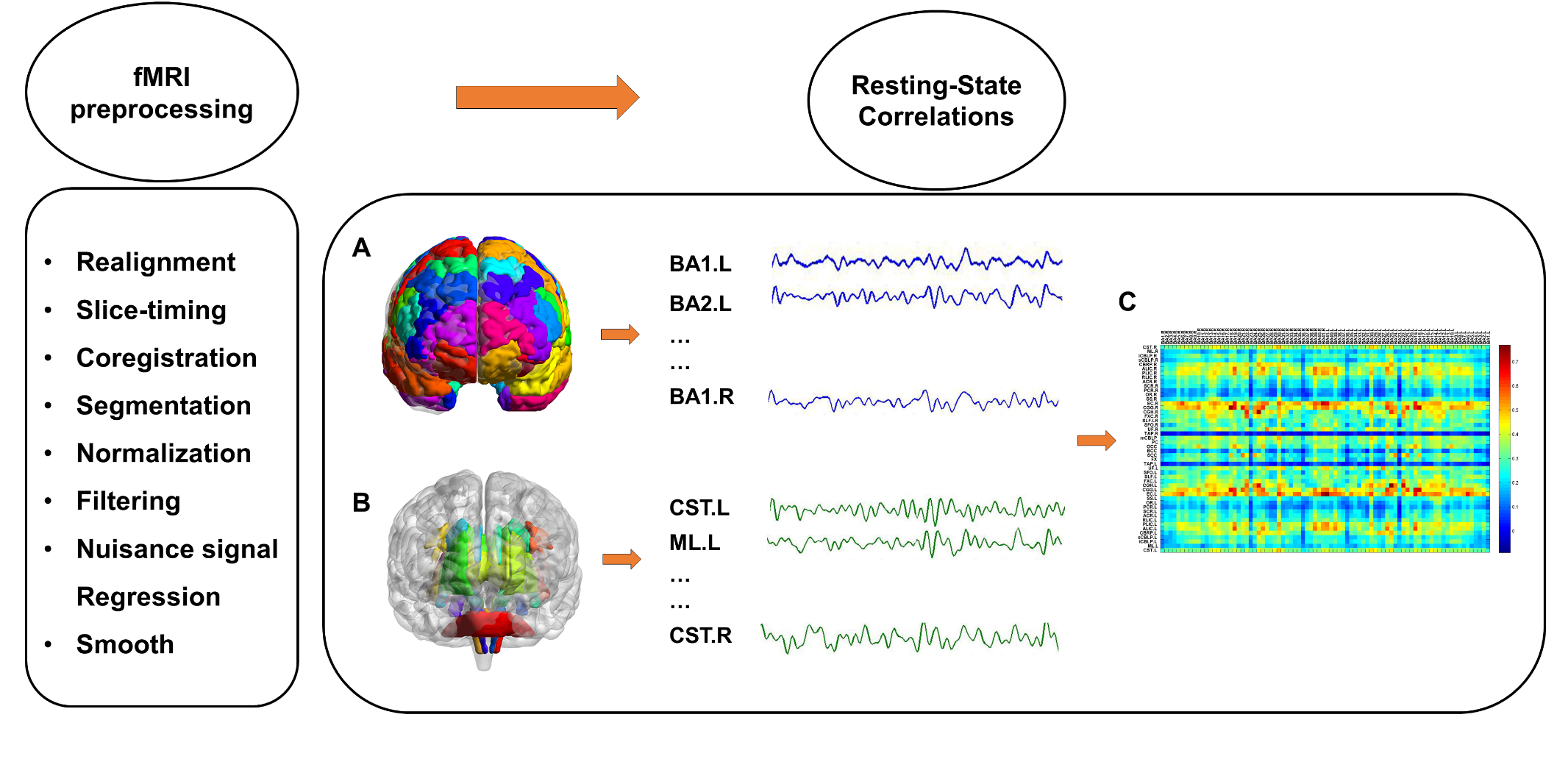


**SFigure 1**. The pipeline of fMRI data analysis including data preprocessing and computing functional connections between each of the WM bundles and GM regions. Left: fMRI data preprocessing steps. Right: (A) Extracting mean time series from the Brodmann atlas. (B) Extracting mean time series from WM bundles by the JHU IBM-DTI-81 atlas. (C) Functional correlations between GM regions and WM bundles.


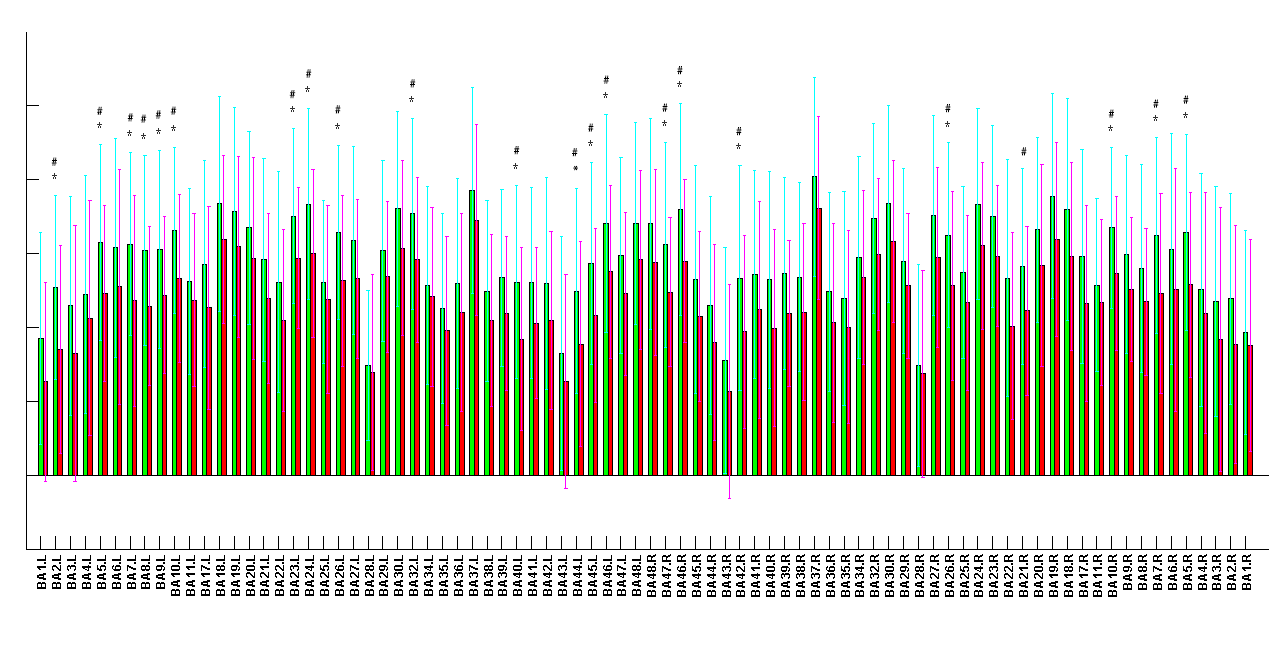


**SFigure 2.** Quantitative comparisons between patients with MWoA and HCs in WM-averaged correlation coefficients of each GM region. The asterisks (*) denote p< N/1(0. 0.012195). The (#) denote p (FDR adjusted) <0.05.


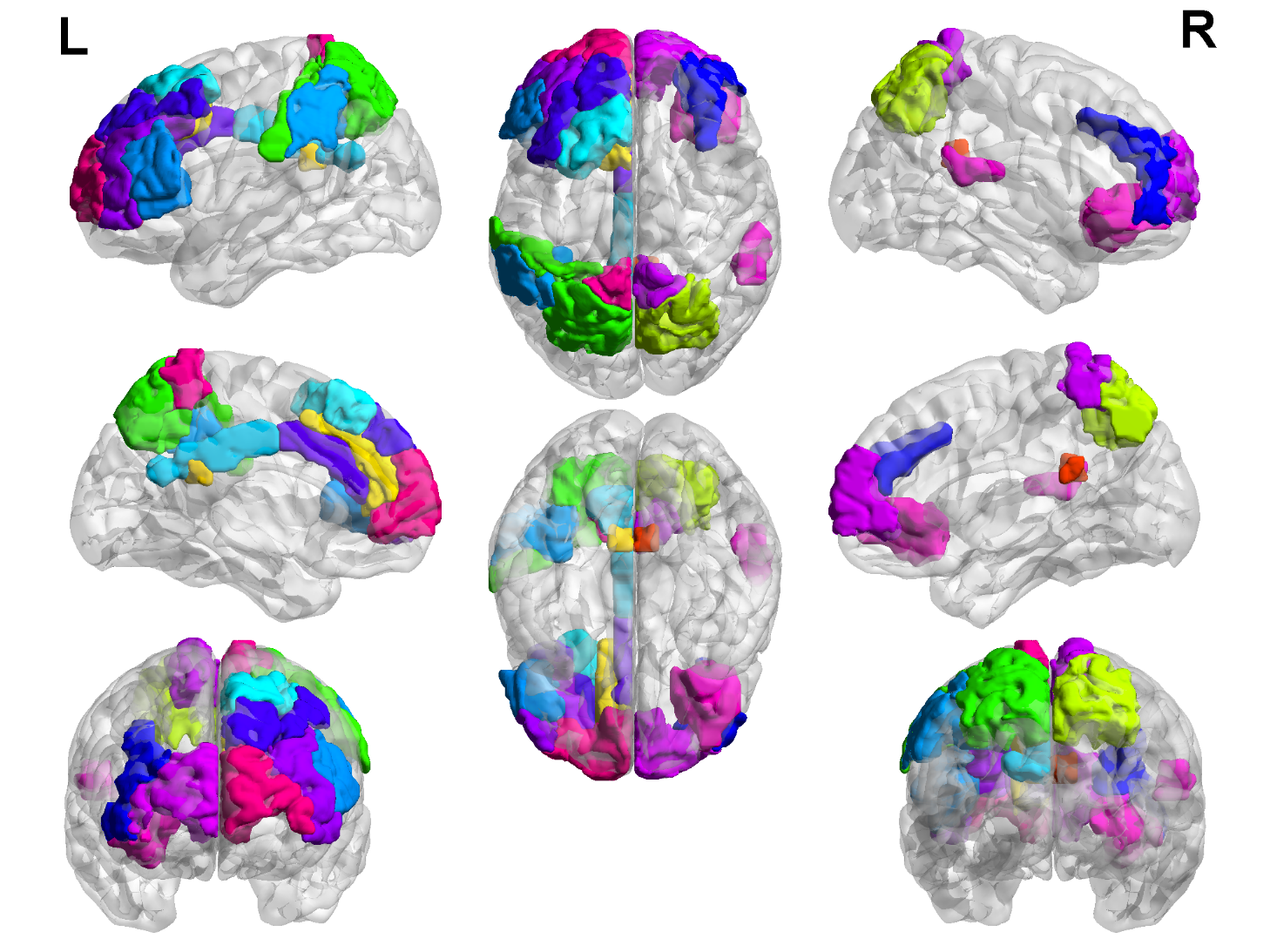


**SFigure 3.** GM regions in the cerebrum of MNI space with decreased WM-averaged correlation coefficients.
